# Genome-Wide Profiling of miRNAs and Other Small Non-Coding RNAs in the *Verticillium dahliae*–Inoculated Cotton Roots

**DOI:** 10.1371/journal.pone.0035765

**Published:** 2012-04-25

**Authors:** Zujun Yin, Yan Li, Xiulan Han, Fafu Shen

**Affiliations:** 1 State Key Laboratory of Crop Biology, College of Agronomy, Shandong Agricultural University, Tai'an, Shandong, China; 2 State Key Laboratory of Cotton Biology, Cotton Research Institute, Chinese Academy of Agricultural Sciences, Anyang, Henan, China; Korea University, Republic of Korea

## Abstract

MicroRNAs (miRNAs) and small interfering RNAs (siRNAs) are short (19–25 nucleotides) non-coding RNA molecules that have large-scale regulatory effects on development and stress responses in plants. Verticillium wilt is a vascular disease in plants caused by the fungal pathogen *Verticillium dahliae*. The objective of this study is to investigate the transcriptional profile of miRNAs and other small non-coding RNAs in Verticillium–inoculated cotton roots. Four small RNA libraries were constructed from mocked and infected roots of two cotton cultured species which are with different Verticillium wilt tolerance (‘Hai-7124’, *Gossypium barbadense* L., a Verticillium-tolerant cultivar, and ‘Yi-11’, *Gossypium hirsutum* L. a Verticillium-sensitive cultivar). The length distribution of obtained small RNAs was significantly different between libraries. There were a total of 215 miRNA families identified in the two cotton species. Of them 14 were novel miRNAs. There were >65 families with different expression between libraries. We also identified two trans-acting siRNAs and thousands of endogenous siRNA candidates, and hundred of them exhibited altered expression after inoculation of Verticillium. Interesting, many siRNAs were found with a perfect match with retrotransposon sequences, suggested that retrotransposons maybe one of sources for the generation of plant endogenous siRNAs. The profiling of these miRNAs and other small non-coding RNAs lay the foundation for further understanding of small RNAs function in the regulation of Verticillium defence responses in cotton roots.

## Introduction

MicroRNAs (miRNAs) are small, non-coding RNAs known for their roles in regulating gene expression by cleaving or inhibiting the translation of target gene transcripts [1], [2]. They can be encoded in their own genes, which are transcribed by RNA polymerase II or exist in introns of protein-coding genes. Primary transcripts of miRNAs are processed to give rise to short 20- to 24-nt-long mature miRNAs. Many plant miRNAs identified in the early studies are conserved across species boundaries [3]. Recent advances in high-throughput sequencing methods have revolutionized the identification of low-abundance non-conserved miRNAs. Moreover, several other classes of small regulatory RNAs, distinguished by their origin and biological function, have also been identified in recent years. These include small interfering RNAs (siRNAs), encompassing chromatin-associated siRNAs and trans-acting siRNAs (tasiRNAs), repeat-associated siRNAs (rasiRNAs), and natural antisense transcript (NAT)-associated siRNAs (nat-siRNAs). In general, miRNAs are generated from single stranded (ss) hairpin RNA precursors by an RNase III type enzyme Dicer-like (DCL) 1 and/or DCL4. Other endogenous siRNAs are processed from long double-stranded RNAs and often require RNA-dependent RNA polymerases (RDRPs or RDRs) and plant-specific nuclear RNA polymerase (Pol) IV or V [4], [5], [6].

Small RNA-mediated gene silencing is a conserved regulatory mechanism underlying plant development, metabolism, and responses to biotic and abiotic stresses. Although antiviral defense mediated by virus-derived small RNAs has been well studied in both plants and animals [7], endogenous small RNAs function in plant immunity have only been demonstrated recently. The first miRNA found to contribute to plant immune systems was the miR393 in *Arabidopsis thaliana* [8]. The miRNA was induced by a bacterial PAMP peptide flg22 and negatively regulated transcripts of a number of F-box auxin receptors. In addition to miR393, miR167 and miR160 that target auxin response factors (ARF) were also induced by bacterial pathogen *Pseudomonas syringae* [9], [10]. Katiyar-Agarwal *et al* [11] identified an endogenous nat-siRNA, nat-siRNAATGB2, which is specifically induced by a virulent *P. syringae* carrying AvrRpt2 (*avrRpt2*). This siRNA contributed to RPS2-mediated bacteria resistance by repressing a putative negative regulator of the *RPS2* resistance pathway. During searching for pathogen-regulated small RNAs, a novel class of 30- to 40-nt-long endogenous siRNAs, lsiRNAs was identified [12]. The *avrRpt2*-induction of AtlsiRNA1 down-regulated the antisense gene AtRAP by destabilizing the target mRNA through decapping and XRN4-mediated 5′-3′decay. In addition to *P. syringae*, there were also many novle miRNAs and other small RNAs which involved in legume-*Rhizobium* symbiosis [13] and the response to the infection of Cyst nematodes [14].

*Verticillium dahliae* Kleb. is a soilborne fungal pathogen causing vascular wilt of more than two hundred dicotyledonous plant species, including cotton (*Gossypium* spp.) [15]. Vascular wilts are particularly notorious since, the pathogens colonize in the vascular system of host plants and few fungicides exist to cure plants once they are infected. To test whether small RNA-mediated gene silencing is involved in plant defence against Verticillium genus, various components of RNA-silencing pathways has been tested in Arabidopsis mutants [16]. As many as ten different components, namely AGO7, DCL4, NRPD1a, RDR2, SGS1, SGS2/RDR6/SDE1, SGS3, AGO1, HEN1, and HST were shown to affect Verticillium-specific defence, suggesting that multiple RNA-silencing pathways play significant roles in regulation of the pathogen defence responses. To further study the regulatory process, present step is to global identify small RNAs in the Verticillium–infected organisms. Cotton is the most important natural fiber crop in the world. Variation in tolerance to Verticillium wilt has been observed among different cultivars. Sea-island cotton (*Gossypium barbadense* L.) is reported to be more tolerant to Verticillium wilt than many other cotton cultivars [17], [18]. Whereas, upland cotton (*Gossypium hirsutum* L.), the mainly cultivar on a large scale, is sensitive to the pathogen [17]. In this study, the two cotton species were used as models. The objective of our study is to investigate the transcriptional profile of miRNAs and other small non-coding RNAs in the Verticillium–inoculated cotton roots.

## Results

### An overview of the sequencing results

Four separate cDNA libraries of small RNAs (sRNAs) were generated from cotton roots including two from *G. hirsutum* (Gh-mock: mock-infected; Gh-inft: Verticillium-infected) and two from *G. barbadense* (Gb-mock: mock-infected; Gb-inft: Verticillium-infected). The sRNA digitalization analysis based on Solexa sequencing takes the SBS-sequencing by synthesis. Solexa raw data were available at Gene Expression Omnibus [GEO: GSE28236].

**Figure 1:**
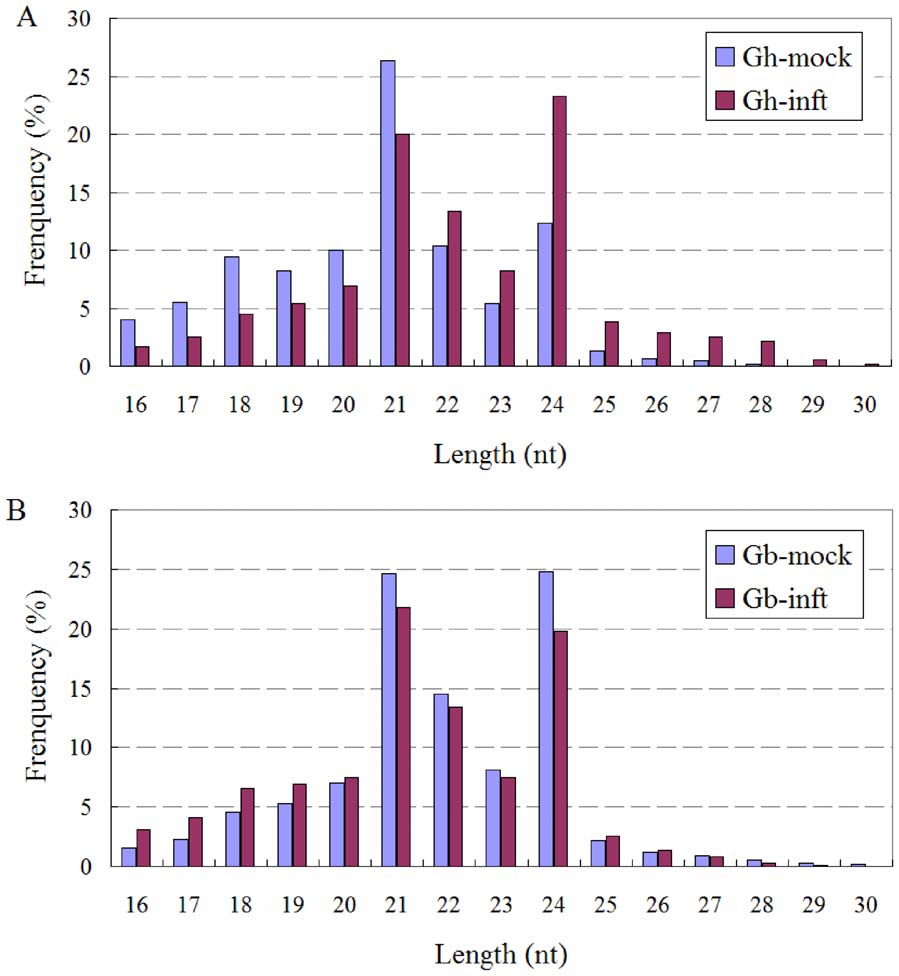
The length distribution of small RNAs in *G. hirsutum* roots (A) and *G. barbadense* roots (B). Gh-mock: mock-infected *G. hirsutum* roots; Gh-inft: Verticillium-infected *G. hirsutum* roots; Gb-mock: mock-infected *G. barbadense* roots; Gb-inft: Verticillium-infected *G. barbadense* roots.

After removing the reads of low quality and masking adaptor sequences, approximately 15,000,000 alignable small RNA reads were obtained in each library (Table 1). To simplify the sequencing data, all identical sequence reads in each sRNA library were grouped and converted into unique sequence tags with associated counts of the individual sequence reads. There were 519,162 (in Gh-mock), 479,724 (in Gh-inft), 561,635 (in Gb-mock), and 496,455 (in Gb-inft) unique tags in the four sRNA libraries. Although the total numbers of unique tags were approximately the same, the fractions of these tags were substantially different between libraries. The sample-specific tags accounted for about 40% of all unique sequence reads in each one of the four libraries (Figure S1). To gain insight into the compositions of small RNA pools, their length distribution were subjected to analyzed. Approximately 75% were 20–24 nt in length with 21 or 24 nt as the major size classes (Figure 1), consistent with being products of cleavage by DCL enzymes. The distribution of different size sRNAs was strikingly different between the two *G. hirsutum* libraries. For Gh-mock data set, the sRNA distribution showed a major peak at 21 nt (26.37%), and another minor peak at 24 nt (12.37%). Instead, the major peak in Gh-inft was at 24 nt (23.25%), and secondary class was 21 nt (19.99%). To exclude the effect from Verticillium sRNAs, the length distribution of sRNAs which specific in Gh-inft library was further analyzed. They could not fill up the gaps between the two datasets of *G. hirsutum* (Figure S2). In *G. barbadense*, the frequency of 21-nt and 24-nt sRNAs showed resemblance, but both were reduced after inoculation of Verticillium. In all, these observations highlighted differences in the complexity of the four small RNA pools, and suggested different regulation underlying the response to Verticillium infection.

**Table 1:** Summary statistics of small RNAs sequenced from roots.

Category	No. of reads			
	Gh-mock	Gh-inft	Gb-mock	Gb-inft
Total reads	19,386,849	17,966,150	17,520,063	16,523,950
High quality	18,664,650	17,037,534	17,520,063	15,747,926
Clean reads	15,635,120	15,747,908	15,599,325	13,720,298
Unique sRNAs	519,162	479,724	561,635	496,455

### Identification of conserved and novel miRNA genes

To investigate the repertoire of conserved miRNAs in *G. hirsutum* and *G. barbadense*, all tag sequences in four sRNA libraries were aligned with all known miRNAs in the central miRNA Registry Database, miRBase database (release 15.0). To minimize false positives, only the unique tags which were represented by more than 2 reads were considered to be *bona fide* miRNAs. As a result, 121 unique tags were found perfectly matching 40 known miRNA families in Gh-mock dataset. There were a total of 4,502 tags identified shorter/longer or contained up to two mismatches to the same 40 and another 93 known miRNA families. Similar to the observation in Gh-mock sRNA library, 124 miRNAs families were identified in the Gh-inft dataset. Among them, 115 members of 38 miRNA families were perfectly match known miRNAs in the miRBase database. In addition, we identified 130 and 121 families in the Gb-mock and Gb-inft library, respectively. Among these miRNA families, 127 miRNAs were expressed in at least two of our four small RNA libraries, and 81 miRNAs were detected in all four sRNA libraries. In this study, we also tried to identify the precursor sequences for these conserved miRNAs. However, due to the fact that the cotton genome has not been fully sequenced, the pre-miRNAs and their secondary structures were only identified for 15 miRNAs.

EST analysis was an economically feasible alternative for miRNA discovery in species lacking a genome sequence [19]. To predict novel miRNAs, the sequencing tags that were annotated as non-coding RNAs were firstly removed. The remaining sequences were mapped to EST sequences to predict novel miRNAs using the MIREAP program. As a result, 45 candidates were detected. For 14 of them, the complementary miRNA* species were cloned in libraries, providing an indication of precise excision from the stem–loop precursor, which has recently been proposed as primary criterion for confident plant miRNA annotation [20]. The lengths of these potential novel miRNAs were 21, 22 or 23 nt, and a half of them began with a 5′ uridine (Table 2). Each miRNA-precursor had a high minimal folding free energy index (MFEI) values (from 0.72 to 1.94 with an average of about 1.12). The value was significantly higher than that for tRNAs (0.64), rRNAs (0.59) and mRNAs (0.62–0.66), as described by Zhang et al [21]. In order to predict potential regulatory targets of these novel miRNAs, a search was performed on cotton mRNA dataset as described in the methods section. 13 putative targets for 7 novel miRNAs were predicted (Table S1). These targets included genes involved in metabolism, protein modification, and RNA or carbohydrate binding, but no transcription factors were identified.

**Table 2:** List of the 14 novel miRNAs in cotton.

Name	Mature miRNA Sequence	Gene ID	LM (nt)	Arm	LP (nt)	G+C (%)	miRNA* Sequence	MFEIs
miR1321	UCUUGGAUCGGACUGGAUUUG	CO117073	21	3′	106	0.37	AAUUCAGUUUGAGCCGAGAUU	1.10
miR1322	UCAAUUUGAUGGAAAUAGAUG	CO080321	21	3′	105	0.44	UCUAUUUUCACUUAAUUGACC	1.18
miR1323	AUAAAAUACUGAUGUGACAUA	CO126815	21	5′	87	0.44	UGUCACGUUAGUAUUUUAUGU	1.94
miR1324	UAGAAAUGGAUGGAAUUUUUA	CO105291	21	3′	139	0.29	AAAAUUUCAUCCAUUUUUAUU	1.40
miR1325	CGAAAACUGUGGCCAAUUUAUU	CO120076	22	3′	261	0.38	UAAAUUGCCACAAUUUUCAAC	0.87
miR1326	UAUACAUUAGAUCAAAGAGCA	CO095028	21	5′	201	0.30	CUCUUUGAUCUAACGUAUAGG	1.09
miR1327	UGAAUAUUGUUAAAGUAGAAA	BG446822	21	3′	124	0.29	UCUACUUUAACAAUAUUCAUA	1.21
miR1328	AGCUACCUAUGAGGCACCCUU	BF272386	21	3′	164	0.48	GGGUGUCUCUUAGGUUGCUAA	1.11
miR1329	UUCAGAAACCAUCCCUUCCUU	CO070343	21	5′	107	0.39	GGAAGGAAUGGUUUCUGAAGC	1.27
miR1330	UUACUUUAGAUGUCUCCUUCA	ES816423	21	5′	131	0.39	AGGGAAACAUCUAAAGUAAAC	0.94
miR1331	AUGCCUAUCAUCAGGAGACUCU	DR456842	22	3′	91	0.46	AAGCUGUUGAUGGCCGGCAUGA	0.72
miR1332	AUGCUCGUGCUCUGUUAUGACCU	ES818236	23	5′	76	0.61	GGCCAUGCAGUGCCGACGUCG	0.77
miR1333	CAUGACUUUUAGCGGCGUUUG	BQ405726	21	3′	67	0.37	AGCGUCGCUAAAGGUCAUGAU	1.31
miR1334	AGCAUUUGUGAUGAUUGUGGU	CO108580	21	3′	109	0.43	CACAAGCAUCAUCGGUGCUGG	0.89

LM: length of mature miRNAs; LP: length of precursor; MFEIs: minimal folding free energy indexes.

### MiRNAs expression patterns in four sRNA libraries

**Figure 2:**
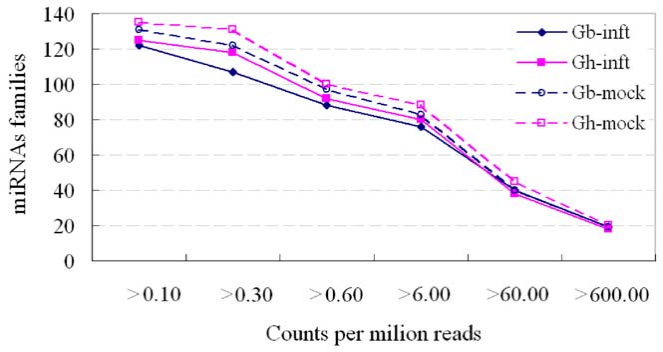
Distribution of miRNA counts over different tag abundance categories from the four libraries.

MiRNAs had a very broad range of expression which varied from hundred thousands of sequence reads for the most abundant miRNAs to some miRNAs did not identified in some samples although they were expressed in other samples. In the total data set, miR156/157, miR166, and miR2947 had a dominant number of reads and were expressed more than 100,000 counts. MiRNAs expression abundance in data sets was analyzed by counting the number of transcripts per million (TPM) clean reads in libraries. The distribution of miRNA counts showed similar tendencies for the four libraries (Figure 2). Among them, less than 21 families had the count number higher than 600 counts per million reads, approximately 65 families were 60–600 counts, and more than 50 families were present between 0.10 and 6.00 counts. The varied frequency of sequencing between miRNA families might suggest their distinct physiological roles in root development.

**Figure 3:**
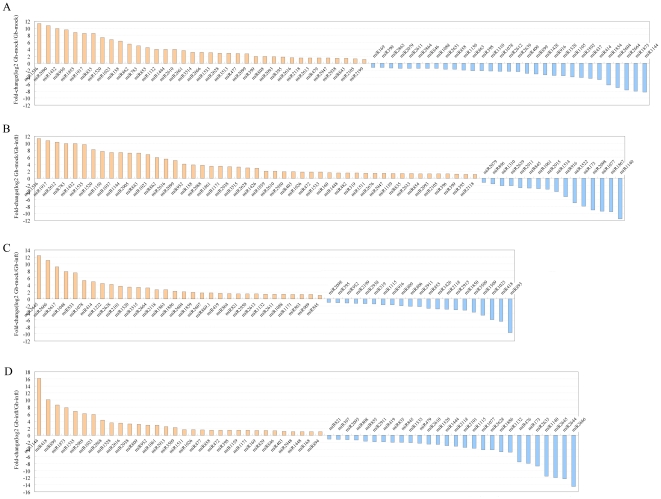
Differentially expressed miRNAs between libraries. Gh-mock: mock-infected *G. hirsutum* roots; Gh-inft: Verticillium-infected *G. hirsutum* roots; Gb-mock: mock-infected *G. barbadense* roots; Gb-inft: Verticillium-infected *G. barbadense* roots.

Differentially expressed miRNAs between libraries give a clue to molecular events related to the root responses to Verticillium infection. We first normalized the read density measurement and then used *P*-value<0.01 and the absolute value of |log_2_^Ratio^|≥1 as a threshold to judge the statistical significance of miRNA expression. From the four data sets, plenty of genes were found to be differentially expressed between libraries. We first made a comparative analysis of miRNA expression between the two cotton species with mock-infected treatment (Figure 3A, Table S2). It was shown that a total of 68 miRNAs had a species-specific expression. Among them, 31 miRNAs were preferentially expressed in *G. barbadense* roots and 37 were preferentially expressed in *G. hirsutum* roots. Notably, there included few well-conserved miRNAs, and mostly were less-conserved miRNAs. The most abundant miRNAs with different expression between two cottons was miR1520, which exhibited high level (read count 53,936) in *G. hirsutum* but less expressed in *G. barbadense* (Table S2). This miRNA were firstly cloned in *Glycine max* using Illumina's SBS sequencing technology, but was unknown for its target genes [22]. Majority of these differently expressed miRNAs had the accumulation about 380 counts. Although their accumulation was not high, they might be playing a significant role in forming genotype-specific phenotype. There were 63 miRNAs that were differently expressed between Gh-mock and Gh-inft libraries (Figure 3B). Among them, 16 miRNAs were of higher abundance and 47 were of lower abundance in Gh-inft library (Table S2), indicating that the expression levels of many miRNAs was suppressed during Verticillium inoculation. In the Verticillium-tolerant *G. barbadense* species, there were also many miRNAs with altered expression in response to Verticillium infection. However, the number of down-regulated miRNAs in Gb-inft library was little fewer than that in Gh-inft library (Figure 3C, Table S2). Five miRNAs were not detected in infected-*G. barbadense* roots, of which miR2666 and miR2645 had a high abundance in mocked roots (read count 333 and 859, respectively). Because of the genotype-specific expression of miRNAs under mock-infected treatment, it was plausible to assume that some miRNAs had preferential expression in one of the two species, when both of them were inoculated by Verticillium. Thus, we compared the expression levels of miRNAs between Gh-inft and Gb-inft libraries. We found that 28 miRNAs were preferentially expressed in Gb-inft and 32 were preferentially expressed in Gh-inft (Figure 3D, Table S2). Among them, less-conserved miRNAs were also the dominating classes, such as miR1026, miR1535, miR1520. To gain insight into possible roles of the differently expressed miRNAs, their target genes were investigated (Table S3). The target genes were classified into dozens of biological processes, including metabolism, transport, catabolic process, response to stress and stimulus, biosynthetic process, regulation, cell communication, and so on. It implied that normal developmental processes were strongly affected after pathogen invasion and many underlay process contribute to the resistance of cotton to disease. In all, these observations suggested that miRNAs play crucial roles not only in general stress responses but also in stress adaptation. Since the tolerant and sensitive varieties have a different regulation in response to the pathogen stress, differently expressed miRNAs between two cotton species may be the key genetic contributors to Verticillium-tolerance trait.

### TAS3 and candidate endogenous siRNAs

**Figure 4:**
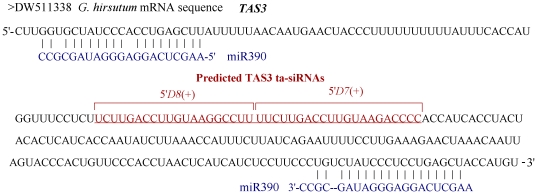
*TAS3*-like locus in cotton. The space size between the two miR390 complementary sites was less than 231 nt (11 phases).

TAS3 and the TAS3-miR390 target sites have been shown high conservation from mosses to seed plants. Using the homology search approach as described by Shen et al [23], *TAS3a*, with two 21-nt tasiARFs, was identified in libraries (Figure 4). This TAS transcript had perfect hits in four cotton EST sequences, DW511338, DW503626, CO077318 and CO077320. In their sequences, two miR390 target sites were found, and the space between the two sites was less than 273 nt (13 phases). The identical D7 and D8 sequences suggested their high conservation between Arabidopsis and cotton. Examination of their expression showed that there was a distinct, narrow peak at the position of the *D7*(+) *TAS3* sequence (Table 3). While the *D8*(+) sequence was weakly expressed. It seems that the less abundant siRNA produced was not functional siRNA. The expression level of *D7*(+) *TAS3* was reduced in infected-*G. hirsutum* roots, suggesting its involvement in the response to Verticillium infection.

**Table 3:** Abundance of two trans-acting siRNA genes (*TAS3*).

name	sequences	No. of reads			
		Gh-mock	Gh-inft	Gb-mock	Gb-inft
*TAS3 D8*(+)	UUCUUGACCUUGUAAGGCCUU	7	6	9	6
*TAS3 D7*(+)	UUCUUGACCUUGUAAGACCCC	558	249	480	342

Of the sRNAs data set, a large portion was found to be able to form a 20–25 nt double-strand RNA duplex, each strand of which was 2 nt longer than the other on the 3′ end. Due to the fact that these sRNAs showed similar structural signatures to siRNA transcripts reported in Arabidopsis and *Oryza sativa* [24], [25], the small RNA transcripts that were mapped onto the antisense strand of cotton EST sequences were regarded as candidate endogenous siRNAs (endo-siRNAs). SiRNAs are typically considered to be less conserved than miRNAs. So, small RNAs in *G. hirsutum* and *G. barbadense* libraries were aligned to their corresponding EST sequences in NCBI. After alignment, we identified 12,709 and 10,824 unique tags in Gh-mock and Gh-inft library, respectively. Due to the small content of *G. barbadense* EST sequences in NCBI, only 2435 and 1981 unique tags were identified in Gb-mock and Gb-inft library, respectively. The proportion of endo-siRNAs in libraries was much lower than that of miRNAs. However, because of incomplete mRNA sequence information in the cotton EST database, the actual number of endo-siRNAs was likely to be higher. The length of endo-siRNAs showed a major peak at 21 nucleotides. The A, U, C have a similar percentage in their 5′ end (∼28%). This was significantly different from the feature of miRNAs with a strandard 5′ terminus. Further analysis was performed to compare their expression abundance between libraries. There were hundreds of siRNAs specially expressed in one of the two *G. barbadense* libraries (Table S4). Besides, 40 siRNAs were found to be suppressed in response to Verticillium infection, and 14 were induced. In *G. hirsutum*, 183 siRNAs exhibited up-regulated expression in infected roots, but 514 were down-regulated (Table S5). These observations suggested that in addition to the regulation from miRNAs, endo-siRNAs should also play a significant role in the response to Verticillium infection.

### Some small RNAs with perfect match with retrotransposons

**Figure 5:**
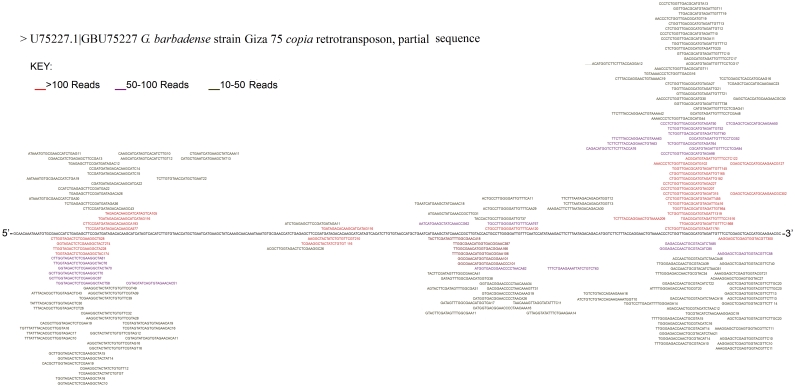
The small RNAs with a perfect match to U75227.1|GBU75227 *G. barbadense* strain Giza 75 *copia* retrotransposon. The color represents abundance of the sequences.

There are 5578 Gossypium retrotransposon elements deposited in NCBI. They were obtained by amplifying their reverse transcriptase (RT) regions, and were used to analyze genome size variation of cotton [26], [27], [28]. Herein, many small RNAs which could form RNA duplex were found with a perfect match with retrotransposon sequences (Figure 5). There were 112,534, 74,234, 123,787, 100,146 reads in Gh-mock, Gh-inft, Gb-mock, and Gb-inft library, respectively. Among these small RNAs, majority (65%) were mapped onto three retrotransposons sequences, U75227, DX402782, and DX404336. To further analyze their characterization, secondary structures of the three retrotransposons were predicted. However, no long stem-loop structure was generated in their sequences; large loops and breaks fulfilled in the secondary structures. After further analyze the small RNAs in retrotransposons sequences, we found that they could match not only the sense strands but also the antisense strands (Figure 5). So it seems that these small RNA duplexes were not processed from single RNA strand, but might derive from double complementary transcripts of retrotransposons. For each data set of libraries, the distribution of these sRNAs showed a major peak at 21 nt, and another minor peak at 20 nt or 22 nt (Figure S3). The 20 nt and 21 nt classes exhibited little down-regulated expression after Verticillium infection, especially in *G. hirsutum* roots. To study their genetic regulatory, the main challenge is verification of the prediction that they are generated from retrotransposons. And related researches are in progress to study this interesting phenomenon.

## Discussion

A global survey of small RNAs in Verticillium–infected organisms will facilitate our understanding of the function and regulatory mechanisms of small RNAs in Verticillium–defence response. In this study, high-throughput sequencing was performed to identify small RNAs that expressed in Verticillium–inoculated cotton roots. This sequencing technique provided a good chance for us to obtain a direct digital readout of small RNAs and achieved an essentially dynamic range of expression between libraries. Two cultivated cotton species, *G. hirsutum* and *G. barbadense*, were used as samples. Both of them are in the AD allotetraploid genomic group, subgenus Karpas Rafinesque [29]. However, they have different tolerance to Verticillium wilt. Comparison of their sequencing data showed that the distribution of different size sRNAs was strikingly different between them. They had a similar distribution on 21-nt sRNAs under mocked treatment. But *G. barbadense* had another preferenclly distribution to 24-nt sRNAs. In small RNA kingdom, RDR2 is a key component for the biogenesis of endogenous 24-nt siRNA [30], [31]; HEN1 methylates small RNA species and thus protects these sRNAs from degradation and polyuridylation [32], [33]; HST mediates the transport of miRNAs from the nucleus to the cytoplasm [34], [35]. Previously, several Arabidopsis mutant which implicated in different RNA-silencing pathways have been used to investigate the biology of sRNAs in response to this vascular wilt pathogen [16]. The mutants *rdr2*, *dcl4*, *rdr6/sde1/sgs2* were found to be more susceptible to Verticillium challenge by showing more severe stunting and necrosis when compared with inoculated wild-type plants. By contrast, the mutants, *hen*, *hst* and *ago1* were found to be more resistant. Based on these observations, it was suggested that the alteration of Verticillium susceptibility does not comply with one single RNA-silencing pathway. In our study, the distribution of kinds of small RNA classes vivid exhibited the regulatory underlying of epigenetics adjustment. The 21-nt and 24-nt sRNAs had a different distribution not only between the two materials, but also between different treatments with Verticillium infection. Therefore, our observations provided an explicit evidence to support the notion that cross-interaction of multiple RNA-silencing pathway involved in Verticillium–defence response.

On the basis of their precursor structures and biogenesis, small RNAs can be divided into miRNAs and small interfering RNAs (siRNAs). The best-characterized class of plant sRNAs is miRNAs [36]. In our study, a total of 215 miRNA families were identified in the two cotton species. There included dozens of miRNAs which were previously annotated as species-specific/lineage-specific miRNAs. For example, miR1917 was firstly reported in *Lycopersicon esculentum* [37]. No homologous to this miRNA was found in the complete genome sequences of Arabidopsis, rice, and *Populus trichocarpa* in previous research. However, we found its expression in cotton, and found that it was high suppressed in infected *G. hirsutum* roots. Many novel and nodulation-regulated miRNAs in *Glycine max* were also found in this study, such as miR2610, miR2612, miR2616 [38]. Evidence for conservation of plant miRNAs has come from genomic and EST sequence data from diverse plants showing sequences containing miRNA hairpins as well as sequences homologous to the known plant miRNAs [19]. To date, >21 families were found in more than 20 plant species, and they were conserved between dicots and monocots, as well as in mosses [39]. In long evolutionary timescales, well-conserved miRNAs have retained homologous target interactions and performed analogous molecular functions across phyla [40]. It is plausible to assume that the conservation is consist with their basically function for normal growth and development of plants. For instance, most conserved miRNAs (miR156, miR159, miR164, miR165/166, miR167, miR169, miR171, miR172, miR319, and miR396) target mRNAs encoding diverse families of transcription factors such as SPLs, ARFs, MYBs, TCPs, NACs, HD-ZIPs, NFY subunits, AP2-like factors, SCLs and GRFs, and the miRNA-guided regulation of these factors is critical for plant development. When the growth and development is stalled under stress, these resources could be mobilized toward adaptive responses to stress.

In our study, >65 miRNAs exhibited altered expression after infection of the fungal pathogen Verticillium. Notably, few well-conserved miRNAs were listed in these pathogen-regulated miRNAs during stress. It suggested that the short time treatment of pathogen stress might not affect growth and development of cotton roots. These pathogen-regulated miRNAs might contribute to species-specific regulation, act as ‘early’ regulators of signal transduction, or might be advantageous for adaptation to stressed environments. In Populus, many miRNAs were found to be induced in abiotic and biotic stressed xylem tissue [41]. A least 11 families were observed in Populus, but were absent from the Arabidopsis genome. Among them, Ptc-miR482, Ptc-miR1444, and Ptc-miR1448 were validated to cleavage PPO genes and disease resistance protein genes, involved in the resistance of plants to biotic and abiotic stresses [42]. Our study showed that these three miRNAs were also conserved in cotton. Besides, miR482 and miR1448 exhibited down-expression after the infection of Verticillium, suggesting their possible roles in acting as positive regulators to response pathogen stress. In tomato, the target genes of miR1917 were validated to be the splice variants of constitutive triple response 4 (*LeCTR4sv1* and *LeCTR4sv2*), which is a member of the CTR family, which are key negative regulators of ethylene responses [37]. Comparative analysis showed that miR1917 showed lower levels of accumulation in normal *G. barbadense* roots, as compared with to normal *G. hirsutum* roots. Otherwise, the accumulation of miR1917 was found to be reduced in responsive to Verticillium infection. In most cases, plant miRNA lead to target mRNAs cleavage. If an miRNA is down-regulated under particular conditions, its target mRNAs should be correspondingly up-regulated coherently. Therefore, it suggested that the expression of miR1917'targets, ethylene signal transduction-related genes, would be up-regulated after pathogen invasion, and function in the cotton defence response. Similarly, miR2118 were also identified as suppressed after pathogen invasion. Its targets were three novel transcripts encoding TIR-NBS-LRR disease-resistance proteins [43]. There were still many other less-conserved miRNAs involved in Verticillium-infection response. However, given the fact that the studies on them are very recent, their functional regulation is not clear. Future analysis on target genes and molecular components downstream could help us to understand their significantly roles in the process in future.

Trans-acting siRNAs (known as *TAS* genes) are a plant specific class of 21-nt endogenous siRNAs that function as miRNA-like posttranscriptional negative regulators [44]. The *TAS3* family is distinguished from other tasiRNA loci by the dual miR390 complementary sites flanking the tasiRNA region and its well conservation [45]. In this study, TAS3a, with two near-identical 21-nt tasiARFs that coaligned with the phases *D7*(+) and *D8*(+), was identified in cotton by homology search. Biological functions have been assigned to the TAS3 family tasiRNA, tasiARF. TasiARF downregulates mRNAs encoding auxin response factors (ARFs), including ARF2, ARF3, and ARF4 [46]. The accumulation of *D7*(+) *TAS3a* sequence were found to be reduced in responsive to Verticillium infection, suggesting that auxin signaling pathway might be involved in this pathology response. In animals, transposable elements (transposons) were found to be one of important sources of endogenous siRNAs. However, little is known regarding the genetic regulation of transposon expression in plants. Until recently, a new class of endogenous siRNAs was found derived from miniature inverted-repeat transposable elements (MITEs) in rice [47] and solanaceae [48]. Can siRNA genes derive from retrotransposons, the class I transposons? Our research found that some small RNAs had a perfect match with retrotransposon sequences. They were able to form a 20–25 nt long-strand RNA duplex, each strand of which was 2 nt longer than the other on the 3′ end. They could match not only the sense strands but also the antisense strands. Take GBU75227 as an example, it is a *G. barbadense* strain Giza 75 *copia* retrotransposon. SiRNAs was not uniformly distributed on its sequence. They were major located at two regions. The space between the two sites was about 160 nt (8 phases). Further analysis found that the retrotransposons could not form standard stem-loop structures. Collectively, these data suggested that the siRNAs were not degraded mRNA templates. Possibly, retrotransposons is one of sources for their generation. Otherwise, we found that the 20 nt and 21 nt classes, which matched to retrotransposons, exhibited an altered expression after Verticillium–infection in *G. hirsutum* roots. Retrotransposons were called ‘selfish’ genes recently, because their only function seems to make more copies of themselves. In Gossypium (cotton), earlier studies suggested that the 3-fold genome size variation among diploids was due largely to copy number variation of retrotransposons [27]. Whiles, our knowledge about retrotransposons is in infant. As more retrotransposon sequence information in the Gossypium becomes available, we expect their genetic regulatory with small RNAs will be identified, which will give greater understanding of the impact of these elements on genome evolution and multiple developmental process of cotton.

In summary, global transcriptional profiles of small non-coding RNAs were investigated in Verticillium–inoculated roots of *G. hirsutum* and *G. barbadense*, two cultured species with different Verticillium tolerance. The different expression patterns of these small RNAs are a valuable resource for further study on posttranscriptional gene regulation in Verticillium–defence response. Accordingly, further identification and detailed kinetics analysis of the target genes of these small RNAs could shed new light on their regulatory roles in this pathology response.

## Materials and Methods

### Plants, pathogen and infection

Two cotton varieties, ‘Hai-7124’ (*G. barbadense*, tolerant) and ‘Yi-11’ (*G. hirsutum*, highly susceptible), were used in this study [18]. The seeds were provided by State Key Laboratory of Crop Biology, China. The sterilized seeds of each variety were put on pasteurized sands for germination. Seedlings were grown in growth chambers at 25–28°C and watered with Hogland culture liquid every 3 days.

A highly aggressive defoliating *V. dahliae* isolate VJ37 from Hebei province in China was used as the pathogen [49]. It was kindly provided by Key Laboratory of Crop Germplasm Resources of Hebei, China. A single spore from the potato-dextrose agar (PDA) plate was inoculated and cultured in Czapek Broth liquid medium for 15 days at 25°C. The conidial suspension obtained was diluted to approximately 10^7^ spores per ml with sterile distilled water before inoculation. Seedlings of each cotton variety with two fully expanded leaves were infected with the pathogen by root-dip inoculation into a suspension of fungal conidia for 5 min, and returned to the Hogland liquid medium for discrete post-inoculation time intervals, as previously described [49]. Control mock-inoculated seedlings were inoculated with water. The roots of both pathogen-infected and mock-infected seedlings were harvested at 12 and 24 hours after inoculation and freezing immediately in liquid nitrogen. The root was chosen as material because the pathogenic fungus infects the roots of plants directly in soil and enters the vessel through the cortical cells [17].

### RNA extraction and small RNA cloning

Total RNA was isolated from roots using a modified CTAB method with isopropanol instead of lithium chloride for RNA precipitation [50]. The total RNAs from roots which were harvested at 12 and 24 hours after inoculation were pooled in an equal fraction ratio. Small RNA cloning was performed as described previously by Sunkar and Zhu [51]. Briefly, 0.5 M NaCl and 10% PEG8000 were used to precipitate and enrich RNAs with low molecular weight. After that, about 100 µg of low molecular weight RNA were processed by 15% denaturing polyacryl amide gel electrophoresis (PAGE). After gel electrophoresis, the small RNA fragments in the range of 10–32 nt were excised and eluted with 0.4 M NaCl overnight at 4°C. The RNA was dephosphorylated using alkaline phosphatase (New England Biolabs, Beijing China) and recovered by ethanol precipitation. The isolated small RNAs were then sequentially ligated to RNA/DNA chimeric oligonucleotide adapters, and were converted to DNA by RT-PCR. Finally, the DNA was sequenced by Solexa sequencing technology (BGI, Shenzhen, China). Solexa sequencing takes the SBS-sequencing by synthesis, is well known for its high through-put and high accuracy with simply operated automatic platform.

### Bioinformatics analysis of small RNA transcriptomes

Individual sequence read with the base quality scores was produced by Solexa. We trimmed the adaptor sequences, filtered out the low quality tags and eliminated contamination of adaptor sequences. The resulted set of the unique sequences with associated read counts was referred as clean sequence tags. For non-coding RNAs identification, clean reads were compared with rRNA, tRNA, snRNA, and snoRNA deposited in NCBI and Rfam database (http://www.sanger.ac.uk/software/Rfam) using the SOAP 2.0 program [52]. Next, sequence tags were searched against the miRNA database, miRBase, in order to identify conserved miRNAs in cotton. A total of 1,763 plant miRNAs belonging to 871 families have been deposited in the miRBase database (release 15.0). All of them were downloaded and were utilized as dataset to perform alignment searches. The reads that were shorter than 24 nt, and had more than one copy in a read library, either shorter/longer or contained up to two mismatches, and no match to known non-coding RNAs, were tested whether they are miRNAs.

To investigate the differentially expressed miRNAs between libraries, firstly, each identified miRNAs read count was normalized to the total number of miRNA reads in each given sample and multiplied by a million. Then, Bayesian method was applied to infer the statistical significance value [53]. This approach was developed for analysis of digital gene expression profiles in previous study and accounts for the sampling variability of tags with low counts. After Bayesian test, if the *P*-value given by this method was <0.01 and the change in normalized sequence counts was more than twofolds, a specific miRNA was considered to be differentially expressed.

Cotton retrotransposon elements were downloaded from the NCBI database. ESTs were downloaded from the NCBI [54] and TIGR database (Release 10.1, http://compbio.dfci.harvard.edu/tgi/plant.html). Small RNA sequences were mapped onto these reference sequences. For monitoring the mapping events on both strands, the sense and antisense sequences were included in the data collection. The small RNA sequences which mapped on ESTs were used to predicted novel potential miRNAs. The selected EST sequences were folded into a secondary structure using the RNAfold program (http://rna.tbi.univie.ac.at/cgi-bin/RNAfold.cgi). The novel miRNAs were identified using the MIREAP program developed by the BGI (Beijing Genome Institute, http://sourceforge.net/projects/mireap/). For this procedures, the occurrence of both a miRNA and a miRNA* in the deep sequencing data was the basis to predict novel miRNAs.

### Target gene prediction

The major steps and parameter settings for predicting target genes of miRNAs were performed as described in previous studies [55], [56], [57]. The miRNA sequences identified were used in a BLAST search against the cotton mRNA database [58]. Sequences with only 0–4 nt mismatches compared with the query miRNA sequences were selected manually. The criteria include allowing one mismatch in the region complementary to nucleotide positions 2 to 12 of the miRNA, but not at position 10/11, which is a predicted cleavage site, and three additional mismatches between positions 12 and 22, but with no more than two continuous mismatches. The G∶U pair was counted as 0.5 mismatch. Because the Unigene database contains many transcript sequences that appear to come from the same transcription locus, and information on protein similarities, data from the Unigene accessions were used to analyze the functional similarity of target genes.

## Supporting Information

Figure S1 **The sample-specific unique sequences from the four libraries.**
(TIF)

Figure S2 **The length size distribution of small RNAs specific in Verticillium-infected** ***G. hirsutum*** **roots.**
(TIF)

Figure S3 **The length distribution of small RNAs that match to retrotransposons perfectly.** (A) The length distribution of small RNAs in *G. hirsutum* roots; (B) The length distribution of small RNAs in *G. barbadense* roots. Gh-mock: mock-infected *G. hirsutum* roots; Gh-inft: Verticillium-infected *G. hirsutum* roots; Gb-mock: mock-infected *G. barbadense* roots; Gb-inft: Verticillium-infected *G. barbadense* roots.
(TIF)

Table S1 **The target genes of novel miRNAs in cotton.**
(DOC)

Table S2 **The relative abundance of differentially expressed miRNAs between libraries.**
(XLS)

Table S3 **Predicted targets of the differentially expressed miRNAs.**
(DOC)

Table S4 **Different expression of candidate endogenous siRNAs between mock- and Verticillium-infected** ***G. barbadense*** **roots.**
(XLS)

Table S5 **Different expression of candidate endogenous siRNAs between mock- and Verticillium-infected** ***G. hirsutum*** **roots.**
(XLS)
