# Supplementary figures and images for: Genome-Wide Profiling of miRNAs and Other Small Non-Coding RNAs in the Verticillium dahliae–Inoculated Cotton Roots
Source: PLoS One. 2012 Apr 25;7(4):e35765. doi: 10.1371/journal.pone.0035765 (PMC3338460; doi:10.1371/journal.pone.0035765)

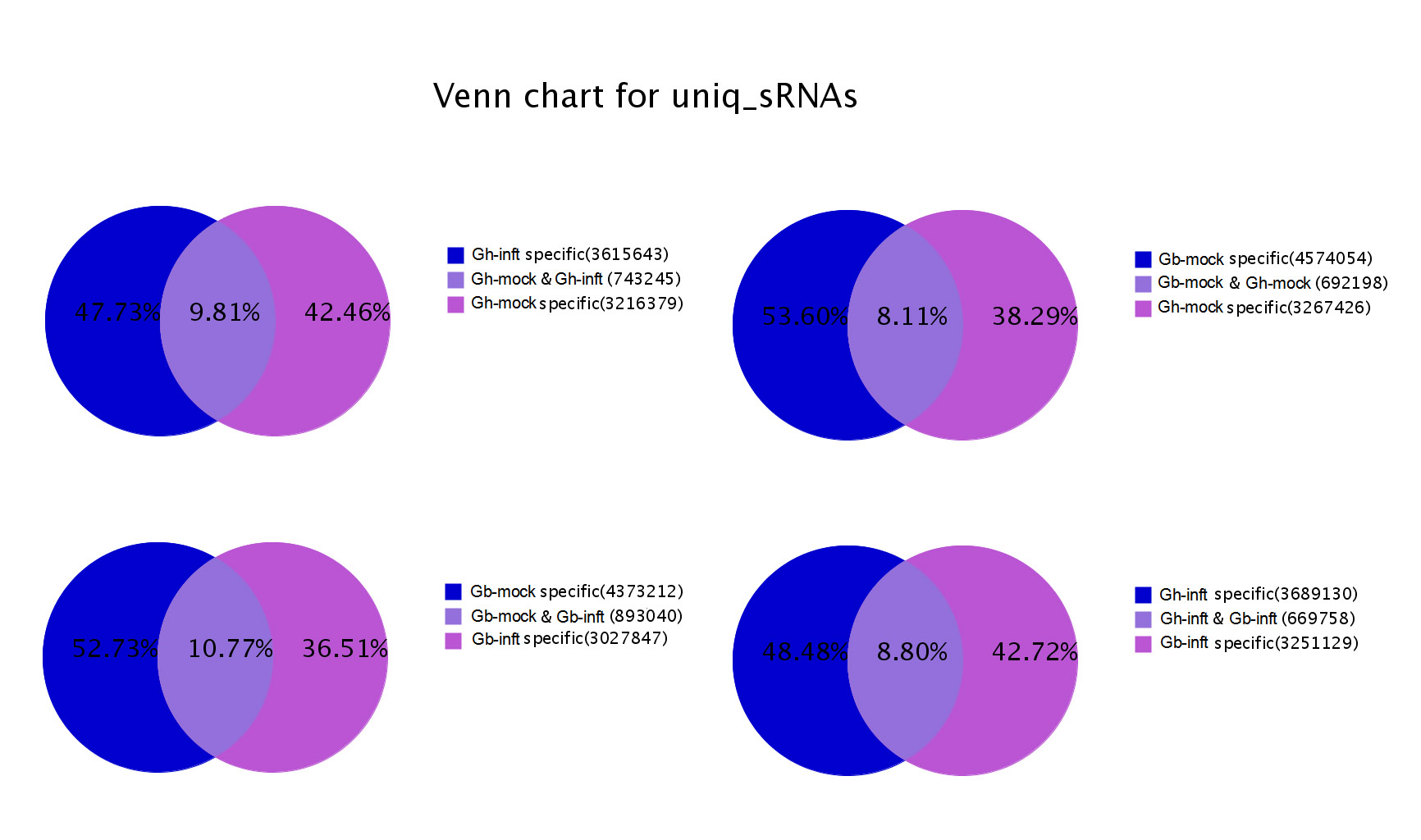

Supplement: Figure S1 — The sample-specific unique sequences from the four libraries. (TIF) [file pone.0035765.s001.tif]

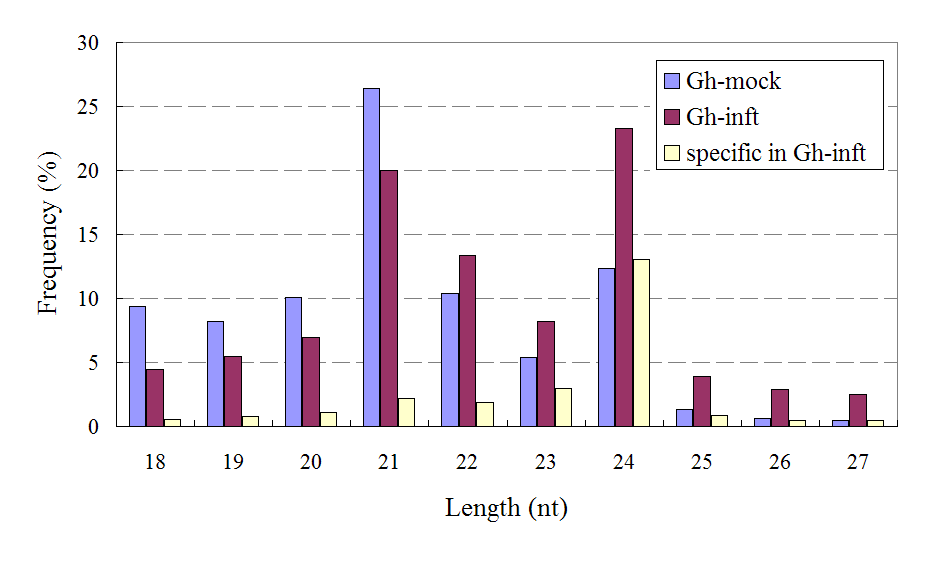

Supplement: Figure S2 — The length size distribution of small RNAs specific in Verticillium-infected G. hirsutum roots. (TIF) [file pone.0035765.s002.tif]

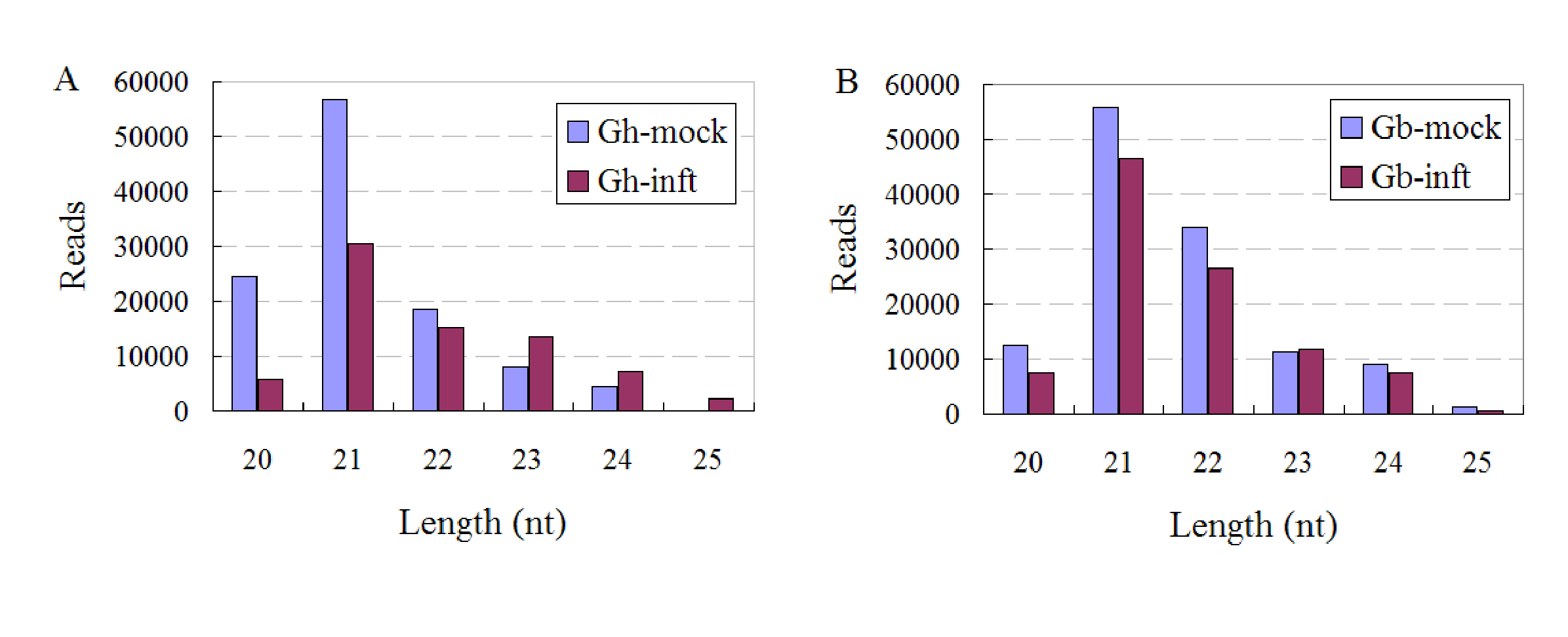

Supplement: Figure S3 — The length distribution of small RNAs that match to retrotransposons perfectly. (A) The length distribution of small RNAs in G. hirsutum roots; (B) The length distribution of small RNAs in G. barbadense roots. Gh-mock: mock-infected G. hirsutum roots; Gh-inft: Verticillium-infected G. hirsutum roots; Gb-mock: mock-infected G. barbadense roots; Gb-inft: Verticillium-infected G. barbadense roots. (TIF) [file pone.0035765.s003.tif]
