# Supplementary material for: Genome-Wide Profiling of miRNAs and Other Small Non-Coding RNAs in the Verticillium dahliae–Inoculated Cotton Roots
Source: PLoS One. 2012 Apr 25;7(4):e35765. doi: 10.1371/journal.pone.0035765 (PMC3338460; doi:10.1371/journal.pone.0035765)
Supplement: Table S1 — The target genes of novel miRNAs in cotton. (DOC) [file pone.0035765.s004.doc]

**Table S1.** The target genes of novel miRNAs in cotton.

| Novel miRNAs | Target genes | Targeted protein |
| --- | --- | --- |
| miR1324 | *Ghi.3553* | nucleotidyltransferase family protein |
| miR1326 | *Ghi.915* | CPK28; ATP binding / calcium ion binding / calmodulin-dependent protein kinase/ protein kinase/ protein serine/threonine kinase |
|  | *Ghi.13327* | GEK1 (GEKO1); D-aminoacyl-tRNA deacylase |
|  | *Ghi.19940* | fumarylacetoacetase, putative |
|  | *Ghi.12192* | DIN4 (DARK INDUCIBLE 4); 3-methyl-2-oxobutanoate dehydrogenase (2-methylpropanoyl-transferring)/ catalytic |
|  | *Ghi.13707* | phototropic-responsive NPH3 family protein |
| miR1329 | *Ghi.9069* | glucan phosphorylase, putative |
|  | *Ghi.10970* | predicted protein |
| miR1321 | *Ghi.16308* | mitotic phosphoprotein N' end (MPPN) family protein |
| miR1331 | *Ghi.24774* | AtSTS (Arabidopsis thaliana stachyose synthase); galactinol-raffinose galactosyltransferase/ hydrolase, hydrolyzing O-glycosyl compounds |
|  | *Ghi.6377* | pyruvate kinase, putative |
| miR1332 | *Ghi.10252* | Unknown protein |
| miR1334 | *Ghi.6533* | amino acid transporter family protein |
