# Supplementary material for: Genome-Wide Profiling of miRNAs and Other Small Non-Coding RNAs in the Verticillium dahliae–Inoculated Cotton Roots
Source: PLoS One. 2012 Apr 25;7(4):e35765. doi: 10.1371/journal.pone.0035765 (PMC3338460; doi:10.1371/journal.pone.0035765)
Supplement: Table S3 — Predicted targets of the differentially expressed miRNAs. (DOC) [file pone.0035765.s006.doc]

**Table S3.** Predicted targets of the differentially expressed miRNAs.

| miRNA family | Fold-change  (log_2_Gh-mock/  Gb-mock) | Target genes | Target description | References |
| --- | --- | --- | --- | --- |
| miR1536 | 11.30 | Glyma07g15330.1 | Deoxyhypusine synthase (EC 2.5.1.46) | Novel and nodulation-regulated microRNAs in soybean roots |
|  |  | [Glyma07g07610.1](http://bioinformatics.cau.edu.cn/cgi-bin/gbrowse/soybean/?name=Glyma07g07610.1) | Chromosome chr15 scaffold_40, whole genome shotgun sequence |  |
|  |  | Glyma02g44920.1 | Ran GTPase binding protein, putative |  |
|  |  | Glyma06g43760.1 | BZIP transcription factor bZIP50 |
|  |  | Glyma09g15360.1 | Beta-galactosidase (EC 3.2.1.23) |
| miR2090 | 10.72 | Os04g25560.1 | serine carboxypeptidase 1 precursor, putative, expressed | PMRD: plant microRNA database |
|  |  | Os04g25560.2 | serine carboxypeptidase 1 precursor, putative, expressed |  |
|  |  | Os09g04810.1 | myosin heavy chain, putative, expressed |  |
|  |  | Os09g04810.2 | myosin heavy chain, putative, expressed |  |
| miR1432 | 9.86 | Os03g59770 | calcium-binding allergen Ole e 8, putative, expressed | Rice MicroRNA Effector Complexes and Targets |
|  |  | Os03g59790 | calcium-binding protein, putative |  |
|  |  | Os03g59870 | calmodulin-like protein, putative, expressed |  |
|  |  | Os04g51610 | calcium-transporting ATPase 9, plasma membrane-type, putative, expressed |  |
| miR950 | 9.57 | No target found | No target found | / |
| miR1855 | 8.76 | Os01g14860 | glycogen synthase kinase-3 homolog MsK-3, putative, expressed | Rice MicroRNA Effector Complexes and Targets |
| miR1917 | 8.54 | TC189700 | LeCTR4sv1 and LeCTR4sv2 | Deep sequencing of tomato short RNAs identifies microRNAs targeting genes involved in fruit ripening |
| miR835 | 8.50 | AT3G61040.1 | CYP76C7 | A diverse and evolutionarily fluid set of microRNAs in Arabidopsis thaliana |
| miR1520 | 7.32 | No target found | No target found | / |
| miR1023 | 6.78 | AT2G13370.1 | Phytochrome-Interacting Protein | High-throughput sequencing of Arabidopsis microRNAs: evidence for frequent birth and death of MIRNA genes |
| miR158 | 6.35 | AT1G64100,AT3G03580 | PPR | ASRP: the Arabidopsis Small RNA Project Database |
| miR1528 | -3.65 | Glyma0041s00340.1 | Q9FEK8 | Ferrochelatase (EC 4.99.1.1) | PMRD: plant microRNA database |
|  |  | Glyma03g25250.1 | B9SDW0 | Glutamate receptor 3 plant, putative |  |
|  |  | Glyma08g35580.1 | C5YZX7 | Putative uncharacterized protein Sb09g023900 |  |
|  |  | Glyma08g41880.1 | B9I9V1 | Predicted protein |  |
|  |  | Glyma08g42640.1 | B9I0D1 | Predicted protein |  |
|  |  | Glyma10g23720.1 | C5YZX7 | Putative uncharacterized protein Sb09g023900 |  |
|  |  | Glyma19g22440.1 | A7PLS7 | Chromosome chr14 scaffold_21, whole genome shotgun sequence |  |
| miR1105 | -3.89 | No target found | No target found | / |
| miR2592 | -4.09 | MTGI9-TC112812 | Glycoside hydrolase, family 47 | Genome-wide Medicago truncatula small RNA analysis revealed novel microRNAs and isoforms differentially regulated in roots and nodules |
|  |  | MTGI9-NP7257119,  MTGI9-NP7272978 | GB|AC144928.17|ABE87821.1 hypothetical protein |  |
|  |  | MTGI9-TC113652 | AAA+ ATPase, core |  |
| miR437 | -4.32 | 11680.m04605; 11680.m04606 | Glu receptor proteins | Cloning and characterization of microRNAs from rice |
| miR414 | -4.65 | contig9329 | URH2 (URIDINE-RIBOHYDROLASE 2); hydrolase [Arabidopsis thaliana] | Cotton EST database |
|  |  | contig9814 | aspartyl protease family protein [Arabidopsis thaliana] |  |
|  |  | contig12913 | TGH (TOUGH); RNA binding / protein binding [Arabidopsis thaliana] |  |
|  |  | contig708 | Os03g0390600 [Oryza sativa (japonica cultivar-group)] |  |
|  |  | contig868 | high mobility group family [Populus trichocarpa] |  |
| miR1854 | -6.16 | contig10455 | ERS (GLUTAMATE TRNA SYNTHETASE); glutamate-tRNA ligase [Arabidopsis thaliana] | Cotton EST database |
|  |  | contig3288 | cytochrome P450 obtusifoliol 14-alpha-demethylase [Populus trichocarpa] |  |
|  |  | contig1216 | hypothetical protein [Vitis vinifera] |  |
|  |  | contig7261 | protein with unknown function [Ricinus communis] |  |
|  |  | contig21591 | pentatricopeptide repeat-containing protein, putative [Ricinus communis] |  |
| miR2604 | -6.93 | MTGI9-ES613250 | unknown | Genome-wide Medicago truncatula small RNA analysis revealed novel microRNAs and isoforms differentially regulated in roots and nodules |
| miR2664 | -7.59 | MTGI9-TC135501 | Concanavalin A-like lectin/glucanase | Genome-wide Medicago truncatula small RNA analysis revealed novel microRNAs and isoforms differentially regulated in roots and nodules |
|  |  | MTGI9-TC118444 | F-box associated type 1 |  |
|  |  | MTGI9-TC129569 | unknown |  |
| miR1873 | -7.89 | Os05g01790 | OsWRKY69 - Superfamily of rice TFs having WRKY and zinc finger domains | Rice MicroRNA Effector Complexes and Targets |
| miR1144 | -8.16 | contig3397 | hypothetical protein [Vitis vinifera] | Cotton EST database |
|  |  | contig9235 | bromodomain protein [Populus trichocarpa] |  |
|  |  | contig13572 | phenylalanine amonnia lyase [Populus trichocarpa] |  |
|  |  | contig3390 | glutathione reductase [Populus trichocarpa] |  |
|  |  | contig10677 | conserved hypothetical protein [Ricinus communis] |  |
|  |  |  |  |  |
| miRNA family | Fold-change  (log_2_Gb-mock/  Gb-inft) | Target genes | Target description | References |
| miR2645 | 12.43 | MTGI9-EX526576 | NB-ARC | Genome-wide Medicago truncatula small RNA analysis revealed novel microRNAs and isoforms differentially regulated in roots and nodules |
|  |  | contig9609 | methyltransferase [Arabidopsis thaliana] | Cotton EST database |
|  |  | contig3196 | hypothetical protein [Vitis vinifera] |  |
|  |  | contig17229 | ADP-ribosylation factor, arf, putative [Ricinus communis] |  |
|  |  | contig12019 | Os07g0564700 [Oryza sativa (japonica cultivar-group)] |  |
| miR2666 | 11.05 | MTGI9-AW688487 | unknown | Genome-wide Medicago truncatula small RNA analysis revealed novel microRNAs and isoforms differentially regulated in roots and nodules |
| miR2617 | 9.15 | No target found | No target found | / |
| miR1048 | 7.85 | Phypa1_1 89542 fgenesh1_pg.scaffold_184000055 | unknown | Common Functions for Diverse Small RNAs of Land Plants |
| miR833 | 7.49 | [AT4G34170.1](http://bioinformatics.cau.edu.cn/cgi-bin/gbrowse/arabidopsis/?name=AT4G34170.1) | kelch repeat-containing F-box family protein | chr4:16368091-16369029 FORWARD | High-throughput sequencing of Arabidopsis microRNAs: evidence for frequent birth and death of MIRNA genes |
| miR1078 | 5.32 | Phypa1_1 86501 fgenesh1_pg.scaffold_146000047 | unknown | Common Functions for Diverse Small RNAs of Land Plants |
| miR414 | 4.90 | contig9329 | URH2 (URIDINE-RIBOHYDROLASE 2); hydrolase [Arabidopsis thaliana] | Cotton EST database |
|  |  | contig9814 | aspartyl protease family protein [Arabidopsis thaliana] |  |
|  |  | contig12913 | TGH (TOUGH); RNA binding / protein binding [Arabidopsis thaliana] |  |
|  |  | contig708 | Os03g0390600 [Oryza sativa (japonica cultivar-group)] |  |
|  |  | contig868 | high mobility group family [Populus trichocarpa] |  |
| miR1222 | 4.40 | AT5G42690.1 | expressed protein, contains Pfam profile PF04784: Protein of unknown function, DUF547; expression supported by MPSS | chr5:17133858-17136351 REVERSE | Aliases: MJB21.6, MJB21_6 | Common Functions for Diverse Small RNAs of Land Plants |
| miR2628 | 4.19 | No target found | No target found | / |
| miR2101 | 3.67 | Os03g51440.1 | ATP binding protein, putative, expressed | PMRD: plant microRNA database |
|  |  | Os11g19460.1 | catalytic/ hydrolase, putative, expressed |  |
| miR893 | -9.55 | No target found | No target found | / |
| miR418 | -6.37 | AT1G26580.1 | similar to myb family transcription factor / ELM2 domain-containing protein [Arabidopsis thaliana] (TAIR:AT2G03470.1); similar to Os03g0425800 [Oryza sativa (japonica cultivar-group)] (GB:NP_001050406.1); similar to expressed protein [Oryza sativa (japonica cultivar-group)] | PMRD: plant microRNA database |
|  |  | AT1G30970.1 | Symbols: SUF4 | SUF4 (SUPPRESSOR OF FRIGIDA4); transcription factor | chr1:11040262-11043732 REVERSE |  |
|  |  | AT1G30970.2 | ymbols: SUF4 | SUF4 (SUPPRESSOR OF FRIGIDA4) | chr1:11040262-11043710 REVERSE |  |
| miR1023 | -5.88 | AT2G16390.1 | SNF2 domain-containing protein / helicase domain-containing protein, low similarity to RAD54 (Drosophila melanogaster) GI:1765914; contains Pfam profiles PF00271: Helicase conserved C-terminal domain, PF00176: SNF2 family N-terminal domain | chr2:7104720-7108178 FORWARD | Aliases: F16F14.11, F16F14_11 | Common Functions for Diverse Small RNAs of Land Plants |
| miR1509 | -4.60 | TC131818 | similar to UniRef100_A0EJF0 Cluster: Beta-glucan-binding protein 1; n=1; Medicago truncatula|Rep: Beta-glucan-binding protein 1 - Medicago truncatula (Barrel medic) | High-throughput sequencing of *Medicago truncatula* short RNAs identifies eight new miRNA families |
| miR3509 | -3.77 | No target found | No target found | / |
| miR1850 | -3.19 | Os04g47410 | metal ion binding protein, putative, expressed | Rice MicroRNA Effector Complexes and Targets |
|  |  | Os10g36650 | actin-2, putative, expressed |  |
|  |  | Os04g33510 | expressed protein |  |
| miR2915 | -3.08 | No target found | No target found | / |
| miR2118 | -2.89 | No target found | No target found | / |
| miR1426 | -2.80 | Os02g02750.1 | ethanolaminephosphotransferase, putative, expressed | PMRD: plant microRNA database |
|  |  | Os04g27210.1 | transposon protein, putative, CACTA, En/Spm sub-class |  |
|  |  | Os05g38710.1 | lipin, N-terminal conserved region family protein, expressed |  |
| miR855 | -2.69 | At2g36400 |  | Genome-wide Medicago truncatula small RNA analysis revealed novel microRNAs and isoforms differentially regulated in roots and nodules |
|  |  | contig25281 | AtPNG1 (Arabidopsis thaliana peptide-N-glycanase 1); catalytic/ peptide-N4-(N-acetyl-beta-glucosaminyl)asparagine amidase | Cotton EST database |
|  |  | contig21174 | Wound-induced protein WIN2 precursor, putative [Ricinus communis] | http://www.leonxie.com/displaymicroRNA.php?id=109 |
|  |  | contig6488 | pyridine nucleotide-disulphide oxidoreductase family protein [Arabidopsis thaliana] |  |
|  |  | contig8726 | PNC1 (PEROXISOMAL ADENINE NUCLEOTIDE CARRIER 1); ADP transmembrane transporter/ ATP transmembrane transporter/ binding [Arabidopsis thaliana] |  |
|  |  |  |  |  |
| miRNA family | Fold-change  (log_2_ Gh-mock/  Gh-inft) | Target genes | Target description | References |
| miR1536 | 11.30 | Glyma07g15330.1 | Deoxyhypusine synthase (EC 2.5.1.46) | Novel and nodulation-regulated microRNAs in soybean roots |
|  |  | [Glyma07g07610.1](http://bioinformatics.cau.edu.cn/cgi-bin/gbrowse/soybean/?name=Glyma07g07610.1) | Chromosome chr15 scaffold_40, whole genome shotgun sequence |  |
|  |  | Glyma02g44920.1 | Ran GTPase binding protein, putative |  |
|  |  | Glyma06g43760.1 | BZIP transcription factor bZIP50 |  |
|  |  | Glyma09g15360.1 | Beta-galactosidase (EC 3.2.1.23) |  |
| miR1917 | 10.72 | TC189700 | LeCTR4sv1 and LeCTR4sv2 | Deep sequencing of tomato short RNAs identifies microRNAs targeting genes involved in fruit ripening |
| miR2612 | 10.27 | MTGI9-TC136396 | Helix-loop-helix DNA-binding | Genome-wide Medicago truncatula small RNA analysis revealed novel microRNAs and isoforms differentially regulated in roots and nodules |
|  |  | MTGI9-TC124287 | Helix-loop-helix DNA-binding |  |
| miR783 | 9.93 | AT2G46260.1 | BTB/POZ domain-containing protein | chr2:19003041-19005706 | PMRD: plant microRNA database |
|  |  | AT4G01090.1 | extra-large G-protein-related | chr4:470515-473792 REVERSE |  |
|  |  | AT1G17850.1 | similar to rhodanese-like domain-containing protein [Arabidopsis thaliana] (TAIR:AT2G40760.1); similar to Os03g0861700 [Oryza sativa (japonica cultivar-group)] (GB:NP_001051982.1); similar to unknown protein [Oryza sativa (japonica cultivar-group)] (GB:AAP44745.1); contains InterPro domain Rhodanese-like; (InterPro:IPR001763) | chr1:6146067-6148843 REVERSE |  |
|  |  | AT1G24310.1 | similar to transporter [Arabidopsis thaliana] (TAIR:AT1G10390.2); similar to Os07g0295400 [Oryza sativa (japonica cultivar-group)] (GB:NP_001059407.1); similar to hypothetical protein DDBDRAFT_0190968 [Dictyostelium discoideum AX4] (GB:XP_646706.1); contains InterPro domain Apolipophorin III-like; (InterPro:IPR011000) | chr1:8624050-8626394 FORWARD |  |
| miR1432 | 9.86 | Os03g59770 | calcium-binding allergen Ole e 8, putative, expressed | Rice MicroRNA Effector Complexes and Targets |
|  |  | Os03g59790 | calcium-binding protein, putative |  |
|  |  | Os03g59870 | calmodulin-like protein, putative, expressed |  |
|  |  | Os04g51610 | calcium-transporting ATPase 9, plasma membrane-type, putative, expressed |  |
| miR1535 | 9.64 | contig4743 | epoxide hydrolase, putative [Ricinus communis] | Cotton EST database |
|  |  | contig10502 | hypothetical protein [Vitis vinifera] |  |
|  |  | contig16061 | predicted protein [Populus trichocarpa] |  |
|  |  | contig15024 | predicted protein [Populus trichocarpa] |  |
| miR1520 | 8.18 | No target found | No target found | / |
| miR1150 | 7.72 | No target found | No target found | / |
| miR1037 | 7.38 | AT5G48600.1 | structural maintenance of chromosomes (SMC) family protein, similar to SP:P50532 Chromosome assembly protein XCAP-C {Xenopus laevis}; contains Pfam profiles PF02483: SMC family C-terminal domain, PF02463: RecF/RecN/SMC N terminal domain | chr5:19719103-19726356 FORWARD | Aliases: K15N18.7, K15N18_7 | Common functions for diverse small RNAs of land plants |
|  |  | Os05g41750.1 | proteinSMC family, C-terminal domain containing protein, expressed |  |
|  |  | AT5G02500.1 | heat shock cognate 70 kDa protein 1 (HSC70-1) (HSP70-1), identical to SP:P22953 Heat shock cognate 70 kDa protein 1 (Hsc70.1) {Arabidopsis thaliana} | chr5:553743-556437 REVERSE | Aliases: T22P11.90, T22P11_90 |  |
|  |  | Os11g47760.1 | proteinHeat shock cognate 70 kDa protein, putative, expressed |  |
| miR1144 | 7.32 | contig3397 | hypothetical protein [Vitis vinifera] | Cotton EST database |
|  |  | contig9235 | bromodomain protein [Populus trichocarpa] |  |
|  |  | contig13572 | phenylalanine amonnia lyase [Populus trichocarpa] |  |
|  |  | contig3390 | glutathione reductase [Populus trichocarpa] |  |
|  |  | contig10677 | conserved hypothetical protein [Ricinus communis] |  |
| miR1061 | -2.98 | AT3G53230.1 | cell division cycle protein 48 | Common functions for diverse small RNAs of land plants |
| miR2915 | -3.11 | No target found | No target found | / |
| miR1514 | -3.77 | TC223409 | Plasticity related 2a-like gene | Novel and nodulation-regulated microRNAs in soybean roots |
|  |  | TC208997,TC219451 | annotation not available | Novel and nodulation-regulated microRNAs in soybean roots |
| miR916 | -5.16 | No target found | No target found | / |
| miR3522 | -6.95 | No target found | No target found | / |
| miR173 | -7.84 | AT2G27400 | TAS1a | AGO1-miR173 complex initiates phased siRNA formation in plants. |
|  |  | AT1G50055 | TAS1b |  |
|  |  | AT2G39675 | TAS1c |  |
|  |  | AT2G39681 | TAS2 |  |
| miR2098 | -8.90 | Os02g38840.1 | glucose-6-phosphate 1-dehydrogenase, cytoplasmic isoform, putative, expressed | PMRD: plant microRNA database |
|  |  | Os02g44510.1 | UDP-glucose glycoprotein glucosyltransferase 1 precursor, putative, expressed |  |
|  |  | Os05g08950.1 | bifunctional protein tilS/hprT, putative, expressed |  |
|  |  | Os06g20070.1 | conserved hypothetical protein |  |
|  |  | Os11g05930.5 | CCT motif family protein, expressed |  |
| miR1077 | -9.24 | No target found | No target found | / |
| miR1867 | -9.42 | Os10g30550 | phosphoglycerate kinase, chloroplast precursor, putative, expressed | Rice MicroRNA Effector Complexes and Targets |
| miR1140 | -11.42 | AT3G61400.1 | 2-oxoglutarate-dependent dioxygenase, putative, similar to 2A6 (GI:599622) and tomato ethylene synthesis regulatory protein E8 (SP:P10967); 1-aminocyclopropane-1-carboxylate oxidase homolog - Arabidopsis thaliana, PIR:S59548 | chr3:22729931-22731372 FORWARD | Aliases: F2A19.2 | / |
|  |  | Os05g08540.1 | proteinoxidoreductase, 2OG-Fe oxygenase family protein, expressed |  |
|  |  |  |  |  |
| miRNA family | Fold-change  (log_2_Gb-inft/  Gh-inft) | Target genes | Target description | References |
| miR1144 | 16.12 | contig3397 | hypothetical protein [Vitis vinifera] | Cotton EST database |
|  |  | contig9235 | bromodomain protein [Populus trichocarpa] |  |
|  |  | contig13572 | phenylalanine amonnia lyase [Populus trichocarpa] |  |
|  |  | contig3390 | glutathione reductase [Populus trichocarpa] |  |
|  |  | contig10677 | conserved hypothetical protein [Ricinus communis] |  |
| miR418 | 10.14 | AT1G26580.1 | similar to myb family transcription factor / ELM2 domain-containing protein [Arabidopsis thaliana] (TAIR:AT2G03470.1); similar to Os03g0425800 [Oryza sativa (japonica cultivar-group)] (GB:NP_001050406.1); similar to expressed protein [Oryza sativa (japonica cultivar-group)] | PMRD: plant microRNA database |
|  |  | AT1G30970.1 | Symbols: SUF4 | SUF4 (SUPPRESSOR OF FRIGIDA4); transcription factor | chr1:11040262-11043732 REVERSE |  |
|  |  | AT1G30970.2 | ymbols: SUF4 | SUF4 (SUPPRESSOR OF FRIGIDA4) | chr1:11040262-11043710 REVERSE |  |
| miR899 | 8.59 | No target found | No target found | / |
| miR1873 | 7.82 | Os05g01790 | OsWRKY69 - Superfamily of rice TFs having WRKY and zinc finger domains | Rice MicroRNA Effector Complexes and Targets |
| miR1535 | 6.86 | contig4743 | poxide hydrolase, putative [Ricinus communis] | Cotton EST database |
|  |  | contig10502 | hypothetical protein [Vitis vinifera] |  |
|  |  | contig12594 | predicted protein [Populus trichocarpa] |  |
|  |  | contig16061 | predicted protein [Populus trichocarpa] |  |
| miR2905 | 6.12 | Os08g30780 | ATATH3,, putative | Rice MicroRNA Effector Complexes and Targets |
|  |  | Os06g39840 | retrotransposon protein, putative, unclassified |  |
|  |  | Os01g14100 | folate/biopterin transporter family protein, expressed |  |
|  |  | Os05g40170 | expressed protein |  |
|  |  | Os02g24134 | vacuolar protein-sorting protein 45, putative, expressed |  |
| miR1023 | 5.90 | AT2G16390.1 | SNF2 domain-containing protein / helicase domain-containing protein, low similarity to RAD54 (Drosophila melanogaster) GI:1765914; contains Pfam profiles PF00271: Helicase conserved C-terminal domain, PF00176: SNF2 family N-terminal domain | chr2:7104720-7108178 FORWARD | Aliases: F16F14.11, F16F14_11 | Common Functions for Diverse Small RNAs of Land Plants |
| miR2868 | 4.31 | contig725 | ATAF1; transcription activator/ transcription factor [Arabidopsis thaliana] | Cotton EST database |
|  |  | contig2904 | multidrug resistance protein ABC transporter family [Populus trichocarpa]; |  |
|  |  | contig3285 | hypothetical protein SORBIDRAFT_07g003600 [Sorghum bicolor] |  |
|  |  | contig3709 | AtMAPR2 (Arabidopsis thaliana membrane-associated progesterone binding protein 2); heme binding |  |
|  |  | contig12265 | chromatin remodeling complex subunit [Populus trichocarpa] |  |
|  |  | contig13265 | zinc finger (C3HC4-type RING finger) family protein [Arabidopsis thaliana] |  |
|  |  | contig21236 | ATRX; ATP binding / DNA binding / helicase/ nucleic acid binding [Arabidopsis thaliana] |  |
|  |  | contig14603 | o-methyltransferase, putative [Ricinus communis] |  |
|  |  | contig17895 | AMP-activated protein kinase, gamma regulatory subunit, putative [Ricinus communis] |  |
| miR1528 | 3.52 | Glyma0041s00340.1 | Q9FEK8 | Ferrochelatase (EC 4.99.1.1) | PMRD: plant microRNA database |
|  |  | Glyma03g25250.1 | B9SDW0 | Glutamate receptor 3 plant, putative |  |
|  |  | Glyma08g35580.1 | C5YZX7 | Putative uncharacterized protein Sb09g023900 |  |
|  |  | Glyma08g41880.1 | B9I9V1 | Predicted protein |  |
|  |  | Glyma08g42640.1 | B9I0D1 | Predicted protein |  |
|  |  | Glyma10g23720.1 | C5YZX7 | Putative uncharacterized protein Sb09g023900 |  |
|  |  | Glyma19g22440.1 | A7PLS7 | Chromosome chr14 scaffold_21, whole genome shotgun sequence |  |
| miR2616 | 3.52 | MTGI9-NP7251560 | GB|AC159124.15|ABO83898.1 hypothetical protein | Genome-wide Medicago truncatula small RNA analysis revealed novel microRNAs and isoforms differentially regulated in roots and nodules |
|  |  | MTGI9-TC140615 | Cytochrome P450 |  |
|  |  | MTGI9-TC123297 | Protein of unknown function DUF794, plant |  |
|  |  | MTGI9-BQ157955 | G-protein coupled receptor, rhodopsin-like |  |
| miR2628 | -4.05 | No target found | No target found | / |
| miR1886 | -4.60 | AT1G02800.1 | CEL2, ATCEL2 | ATCEL2 (Arabidopsis thaliana Cellulase 2); hydrolase, hydrolyzing O-glycosyl compounds | chr1:613216-616191 REVERSE | PMRD: plant microRNA database |
|  |  | AT1G05610.1 | APS2 | APS2 (ADP-GLUCOSE PYROPHOSHORYLASE SMALL SUBUNIT 2); glucose-1-phosphate adenylyltransferase | chr1:1673860-1675933 REVERSE |  |
|  |  | AT2G36870.1 | xyloglucan:xyloglucosyl transferase, putative / xyloglucan endotransglycosylase, putative / endo-xyloglucan transferase, putative | chr2:15479724-15481760 REVERSE |  |
|  |  | AT3G11010.1 | disease resistance family protein / LRR family protein | chr3:3450502-3453689 REVERSE |  |
|  |  | AT5G39820.1 | ANAC094 | ANAC094 (Arabidopsis NAC domain containing protein 94); transcription factor | chr5:15956528-15957719 REVERSE ce family protein / LRR family protein | chr3:3450502-3453689 REVERSE |  |
| miR1132 | -4.75 | contig16554 | hydrolase/ protein serine/threonine phosphatase [Arabidopsis thaliana] | Cotton EST database |
|  |  | contig4626 | sarcosine oxidase [Populus trichocarpa] |  |
|  |  | contig11184 | conserved hypothetical protein [Ricinus communis] |  |
|  |  | contig17751 | ATP binding / protein binding / protein kinase/ protein serine/threonine kinase [Arabidopsis thaliana] |  |
|  |  | contig21156 | dual specificity protein kinase-ttk, putative [Ricinus communis] |  |
| miR476 | -7.47 | eugene3.00131192(0.5),  eugene3.00131188(1),  eugene3.00131190(1),  eugene3.00160204(1),  eugene3.03980005(1),  fgenesh1_pm.C_LG_  XVIII000087(1), eugene3.00061748, eugene3.00062011(1.5), eugene3.00190210(1.5),  eugene3.15500003(1.5), fgenesh1_pg.C_LG_IV000814, fgenesh1_pg.C_scaffold_  1064000001, | PPR | Novel and Mechanical Stress–Responsive MicroRNAs in Populus trichocarpa That Are Absent from Arabidopsis |
| miR173 | -7.84 | fgenesh1_pg.C_scaffold_2400000001, eugene3.00040809, eugene3.00061747, eugene3.00140626, eugene3.00012769, eugene3.00070793,eugene3.00131194, eugene3.00160470 | TAS1a | AGO1-miR173 complex initiates phased siRNA formation in plants. |
|  |  | AT1G50055 | TAS1b |  |
|  |  | AT2G39675 | TAS1c |  |
|  |  | AT2G39681 | TAS2 |  |
| miR2633 | -8.64 | No target found | No target found | / |
| miR1140 | -11.42 | AT3G61400.1 | 2-oxoglutarate-dependent dioxygenase, putative, similar to 2A6 (GI:599622) and tomato ethylene synthesis regulatory protein E8 (SP:P10967); 1-aminocyclopropane-1-carboxylate oxidase homolog - Arabidopsis thaliana, PIR:S59548 | chr3:22729931-22731372 FORWARD | Aliases: F2A19.2 |  |
|  |  | Os05g08540.1 | proteinoxidoreductase, 2OG-Fe oxygenase family protein, expressed |  |
| miR2645 | -11.88 | contig9609 | methyltransferase [Arabidopsis thaliana] | Cotton EST database |
|  |  | contig3196 | hypothetical protein [Vitis vinifera] |  |
|  |  | contig12019 | Os07g0564700 [Oryza sativa (japonica cultivar-group)] |  |
|  |  | contig17229 | ADP-ribosylation factor, arf, putative [Ricinus communis] |  |
|  |  | MTGI9-EX526576 | NB-ARC | Genome-wide Medicago truncatula small RNA analysis revealed novel microRNAs and isoforms differentially regulated in roots and nodules |
| miR2644 | -12.18 | MTGI9-BG648210 | Heat shock protein DnaJ, N-terminal | Genome-wide Medicago truncatula small RNA analysis revealed novel microRNAs and isoforms differentially regulated in roots and nodules |
|  |  | MTGI9-EX528239 | Alcohol dehydrogenase superfamily, zinc-containing GroES-like |  |
|  |  | MTGI9-BG647562 | Expansin 45, endoglucanase-like |  |
|  |  | MTGI9-BG644594 | Calcium-binding EF-hand |  |
|  |  | MTGI9-TC136408 | Nucleotide-binding, alpha-beta plait |  |
|  |  | MTGI9-EX529174 | Transcription initiation factor IIF, beta subunit |  |
|  |  | MTGI9-EX530134 | Glycoside hydrolase, catalytic core |  |
| miR2666 | -14.35 | MTGI9-AW688487 | unknown | Genome-wide Medicago truncatula small RNA analysis revealed novel microRNAs and isoforms differentially regulated in roots and nodules |
